# Harmful Outcomes in Patients Admitted to Yeovil District Hospital in Acute Alcohol Withdrawal

**DOI:** 10.1192/bjo.2022.439

**Published:** 2022-06-20

**Authors:** Elisabeth Germscheid, Thomas Sherwin

**Affiliations:** Yeovil District Hospital, Yeovil, United Kingdom

## Abstract

**Aims:**

Our aim was to assess what proportion of patients in Acute Alcohol Withdrawal (AAW) experience harm during their admission to hospital. Our hypothesis was that patients who came to harm were likely to have had sub-optimal withdrawal management. Therefore, we also aimed to identify any underlying issues in the way AAW is currently managed which may be contributing to harmful outcomes.

**Methods:**

Inclusion criteria for the audit was inpatients at Yeovil District Hospital over a three-month period from May to July 2021, clinically coded under the heading ‘alcohol abuse’, with a minimum two-day admission. Data were gathered from the patients’ medical notes. An outcome was determined as harmful if firstly, it occurred during the withdrawal period, and secondly it was clinically feasible that it had occurred at least in part, as a result of poor AAW management. Notes from 15 patients were qualitatively reviewed, guided by NICE recommendations, to assess both adherence to, and suitability of YDH AWW policy.

**Results:**

Alcohol abuse was identified at the time of medical clerking in all 15 patients. Audit-C scores were completed in 7 patients. All 15 patients had CIWA scoring initiated within 1 hour of clerking, and chlordiazepoxide prescribed as a STAT dose and then a fixed PRN dose according to whether CIWA score was above 10 or not. 10 patients had their CIWA scores monitored for at least 24 hours. 3 out of 15 inpatients had harmful outcomes, including falls, intracerebral haemorrhage, fractured neck of femur, and cardiac arrest.

**Conclusion:**

Overall, adherence to YDH guidelines was good. Despite this, a high proportion of patients admitted under our care were harmed as a result of inadequate management of alcohol withdrawal. Where issues were identified, these were arguably linked to problems with the YDH AAW policy itself. Unclear guidance over how long to monitor CIWA scores, limitation of chlordiazepoxide doses to 10 mg for even the highest CIWA scores, and omission of Audit-C score in the current hospital guidelines, are suggested as contributors to harm in the three patients identified. Going forward, it will be important to review and make appropriate changes to the YDH policy in these areas according to NICE recommendations, to protect our patients from further harm. These results may well have wider implications in terms of adjustment to AAW policy at other hospitals across the UK.

